# Low Serum Creatinine Levels in Early Pregnancy Are Associated with a Higher Incidence of Postpartum Abnormal Glucose Metabolism among Women with Gestational Diabetes Mellitus: A Retrospective Cohort Study

**DOI:** 10.3390/nu15092193

**Published:** 2023-05-05

**Authors:** Nan Chen, Rui Zeng, Changliu Xu, Fenghua Lai, Li Chen, Chenxue Wang, Ling Pei, Zhuyu Li, Yanbing Li, Haipeng Xiao, Xiaopei Cao

**Affiliations:** 1 Department of Endocrinology, The First Affiliated Hospital, Sun Yat-sen University, 58 Zhongshan 2nd Rd., Guangzhou 510080, China; 2 Department of Obstetrics and Gynecology, The First Affiliated Hospital, Sun Yat-sen University, 58 Zhongshan 2nd Rd., Guangzhou 510080, China

**Keywords:** gestational diabetes mellitus, serum creatinine, postpartum glucose metabolism, impaired glucose tolerance, muscle mass, nutrition

## Abstract

The predictive factors for the progression from gestational diabetes mellitus (GDM) to type 2 diabetes remain incompletely elucidated. Our objective was to investigate the link between serum creatinine, a proxy for skeletal muscle mass, and the development of postpartum abnormal glucose metabolism (AGM). Methods: A retrospective review of the medical records of 501 women with GDM was conducted, all of whom underwent a 75 g oral glucose tolerance test (OGTT) between 4 and 12 weeks postpartum. Women were grouped based on quartiles of serum creatinine at the first antenatal visit to estimate the association between serum creatinine and postpartum AGM incidence. Results: Compared with the highest quartile of creatinine, lower quartiles were substantially linked to an increased incidence of postpartum AGM (adjusted odds ratios 3.37 [95% CI 1.77–6.42], 2.42 [95% CI 1.29–4.51] and 2.27 [95% CI 1.23–4.18], respectively). The generalized additive model suggested a linear relationship between serum creatinine levels and the risk of postpartum AGM below 68 µmol/L of serum creatinine levels. A decrease of 2 μmol/L in serum creatinine levels was found to be associated with a 10% increase in the odds of developing postpartum AGM. Linear regression revealed that a low serum creatinine level was linked to a higher postpartum 2-h glucose level and a decreased insulinogenic index (*p* = 0.007 and *p* = 0.027, respectively). Conclusions: An association was observed between lower serum creatinine levels in early pregnancy and an increased risk of postpartum AGM and poorer β-cell function in women with a recent history of GDM. Further research is needed to understand the mechanisms underlying our findings, as well as the role of skeletal muscle mass or nutritional status in early pregnancy on later glucose metabolism.

## 1. Introduction

Gestational diabetes mellitus (GDM), defined as glucose intolerance first identified during the second and third trimesters of pregnancy, affects 3–14% of pregnancies worldwide [1]. Multiple studies have revealed that insulin sensitivity is lowered by roughly 50–60% in the third trimester compared to pre-pregnancy [2,3,4]. GDM develops when a woman’s pancreas fails to secrete enough insulin in response to significantly decreased insulin sensitivity, resulting in varying degrees of hyperglycemia [5]. Women with a prior incidence of GDM are facing a much greater possibility of encountering type 2 diabetes mellitus (T2DM) postpartum, with a 7- to 10-fold increase compared to those who had a normoglycemic pregnancy, and 20–50% of them may develop T2DM within five years postpartum [6,7,8]. Thus, an advisory from the American Diabetes Association advocates that all women with GDM should be examined by a 75 g oral glucose tolerance test (OGTT) at 4–12 weeks after delivery for the reclassification of glucose tolerance status and T2DM prevention [9].

For the purpose of determining the subgroup of women who are most at risk for developing T2DM, it is essential to determine the risk factors for postpartum abnormal glucose metabolism (AGM) following recent GDM. Identified predictive factors included obesity, Asian ethnicity, family history of diabetes, older maternal age, GDM identified prior to the 24th week of pregnancy, glucose levels and the need for insulin therapy during pregnancy [10,11,12,13]. Multiple studies have indicated that high body mass index (BMI) is a risk factor for GDM in African American, Latina or Caucasian women, while it is a less effective predictor in Asian women [14,15]. The mechanism of those discrepant findings remained unclear.

The characteristic of the postpartum AGM in Asian GDM women was higher post- glucose challenge levels [16,17,18].We know that postprandial hyperglycemia is mainly associated with reduced glucose uptake (mainly representing muscle) combined with glucose-stimulated insulin secretion dysfunction. Skeletal muscle serves as a key player as the major site of postprandial glucose disposal, accounting for about 85% of glucose uptake [19]. It has been observed that the content of skeletal muscle is generally lower in Asians than in Caucasians with similar BMI [20,21]. Therefore, one possible explanation for the higher postprandial glucose levels observed in Asian women is the relatively lower skeletal muscle mass in this population. To date, few studies have investigated the relationship between muscle mass and GDM incidence or early postpartum glucose metabolism disorders [22].

Creatinine, the sole metabolite of creatine, is formed almost entirely in skeletal muscle, and the rate of production is closely tied to total skeletal muscle mass [23]. Serum creatinine serves as a reliable and simple-to-measure surrogate marker of skeletal muscle mass, even in stable individuals undergoing chronic dialysis [24,25,26]. Certain prospective cohort studies have indicated that a positive correlation exists between lower serum creatinine levels and an increased risk of T2DM incidence, particularly in Asian individuals [27,28,29,30]. Thus, it would be intriguing to determine whether there is an association between serum creatine, measured in a simple and rapid blood test, and persisting glucose metabolism issues in GDM-afflicted women. Therefore, our retrospective study was designed to investigate the possible link between low serum creatinine levels in early pregnancy and postpartum AGM incidence among women with GDM.

## 2. Materials and Methods

### 2.1. Ethics Approval and Consent to Participate

In compliance with the Declaration of Helsinki, this retrospective study was authorized by the clinical research and animal trial ethics committees of the First Affiliated Hospital, Sun Yat-sen University (approval number: [2020] 048), and patient consent was waived due to the retrospective, non-interventional study design.

### 2.2. Study Design, Sites, and Participants

A retrospective observational cohort study was put forward. From August 2018 to December 2021, women with GDM whose first antenatal visit was at 10–14 weeks and who underwent an OGTT at 4–12 weeks postpartum at the First Affiliated Hospital, Sun Yat-sen University were eligible to partake in this study. At the point of 24–28 weeks’ gestational age, pregnant women completed an OGTT. Following delivery, all patients diagnosed with GDM were encouraged to do a 75 g OGTT test between 4 and 12 weeks after delivery. Women who met one or more of the following criteria were excluded from the study: (1) hyperglycemia at the first antenatal visit or preexisting diabetes; (2) a serum creatinine level that is greater than the upper reference limit, or renal disease; (3) preexisting chronic hypertension; (4) viral infection or positive carrier status; (5) cancer; (6) connective tissue diseases; (7) uncontrolled endocrine disorders; or (8) multiple gestation. Women with incomplete data and no follow-up examination were also removed. Ultimately, the analysis’s final sample size was 501 women.

### 2.3. Data Collection

To obtain patient data, the medical record system of the hospital was examined. Personal information, age, height, pre-pregnancy weight, pre-pregnancy BMI, gestational weight gain, insulin use, parity, familial T2DM history, medication use, previous GDM history, and other disease history were all gathered. The baseline laboratory test results collected included fasting plasma glucose (FPG), serum creatinine, aspartate transaminase (AST), alanine aminotransferase (ALT), blood urea nitrogen (BUN) and γ-glutamyl transpeptidase (GGT) during the first antenatal visit at 10–14 weeks of gestation. Lipid data from middle pregnancy (24–28 weeks) were also included. Standardized methods were used to analyze blood samples collected in the morning after a minimum 8 h overnight fast. 

### 2.4. Definitions

At 24–28 weeks of gestation, all women had a 75 g OGTT performed as a standard check for GDM (1). In line with the standards set forth by the International Association of Diabetes and Pregnancy Study Groups (IADPSG) criteria [31], the GDM diagnosis was established if any of the three criteria below were fulfilled following a 75 g OGTT: FPG levels ≥  92 mg/dL, 1-h glucose levels ≥ 180 mg/dL, and 2-h glucose levels ≥ 153  mg/dL.

A postpartum follow-up 75 g OGTT, consisting of blood sampling at 0, 30, and 120 min, should be performed on all women with a history of GDM. Insulin levels were also assessed. Postpartum AGM included impaired fasting glucose (IFG; 110 mg/dL ≤ FPG < 126 mg/dL), impaired glucose tolerance (IGT; 140 mg/dL≤ 2-h glucose < 200 mg/dL) or diabetes mellitus (DM; FPG ≥ 126 mg/dL and/or 2-h glucose ≥ 200 mg/dL). 

The insulinogenic index (IGI), derived from the OGTT, was used to assess β-cell function. We evaluated insulin sensitivity using HOMA-IR. Below is a list of each index’s formula:

(1) IGI: Δinsulin (30–0 min)/Δglucose (30–0 min) [32];
(2) HOMA-IR = (fasting insulin * fasting glucose)/22.5 [33].

As an attempt to define dyslipidemia in the second trimester of pregnancy, the recommended values for the maternal serum lipid profile in China were consulted: total cholesterol (TC)  ≥  7.50 mmol/L, triglycerides (TGs)  ≥  3.56 mmol/L, high-density lipoprotein cholesterol (HDL-C)  ≤  1.41 mmol/L, and low-density lipoprotein cholesterol (LDL-C)  ≥  4.83 mmol/L [34]. 

### 2.5. Statistical Analysis

The estimation of required minimum sample size was using software G*Power version 3.1 (Heinrich-Heine-Universität Düsseldorf, Düsseldorf, German). The testing method was F test, the effect size f was set at a medium level of 0.25, type I error (alpha) 0.05, power 0.80, and the independent group number was 4. After calculation, the minimum required sample size of a single group was 45. Considering possible dropout and small effect size, we doubled the sample size of each group to 90. Therefore, at least 360 participants would be included. 

By using one-way ANOVA, normally distributed continuous variables were compared and given as the mean and standard deviation. The Mann–Whitney U test was used to compare non-normally distributed data, which were presented as the median with interquartile range (IQR). The frequencies and percentages of categorical variables were calculated and compared using the Chi-square test. The study population was divided into quartiles according to their baseline serum creatinine levels, and the difference in postpartum AGM incidence between quartiles was evaluated using Che chi-square test. To determine the connection between serum creatinine and the incidence of postpartum AGM, odds ratios (ORs) and 95% confidence intervals (Cis) were generated using logistic regression models. Linear regression worked to identify relations of log-transformed creatinine in serum with log-transformed postpartum fasting glucose, 2-h glucose, insulinogenic index, and HOMA-IR. *p* < 0.05 was deemed to be statistical significance. SPSS version 22.0 (IBM Corp, Armonk, NY, USA) was used for statistical analyses. In addition, the general additive model served to detect the shape of the connection between continuous creatinine and the risk of postpartum AGM; these analyses were carried out with the use of the statistical packages R (The R Foundation; http://www.r-project.org (accessed on 10 September 2020); version 3.6.3) and EmpowerStats (www.empowerstats.net (accessed on 2 July 2022), X&Y solutions, Inc. Boston, MA, USA).

## 3. Results

### 3.1. Baseline Characteristics

The study cohort comprised 501 women with a recent diagnosis of GDM, with a median age of 33 years (IQR, 30–37 years), a median BMI of 21.5 kg/m^2^ (IQR, 19.8–23.4 kg/m^2^), a median serum creatinine level of 42 µmol/L (IQR, 46–50 µmol/L), and a median FPG of 77.4 mg/dL (IQR, 72–82.8 mg/dL) at 10–14 weeks gestation. Table 1 presents baseline parameters of the research population’s demographic, clinical, and metabolic traits during pregnancy per first-trimester serum creatinine quartile. By comparison to women with higher serum creatinine levels, those with lower levels tended to be younger, with lower height and weight before pregnancy and lower BUN in early pregnancy. Other risk factors, such as FPG, ALT, AST, and GGT at baseline and lipid profile at 24–28 weeks, did not differ significantly among the serum creatinine quartiles. Compared with the women in the highest creatinine quartile, those with lower creatinine levels appeared to have higher fasting and 2-h OGTT glucose levels during 24–28 weeks of pregnancy, although this difference was not significant.

### 3.2. Association between Serum Creatinine Quartiles in Early Pregnancy and Postpartum AGM

**Figure 1:**
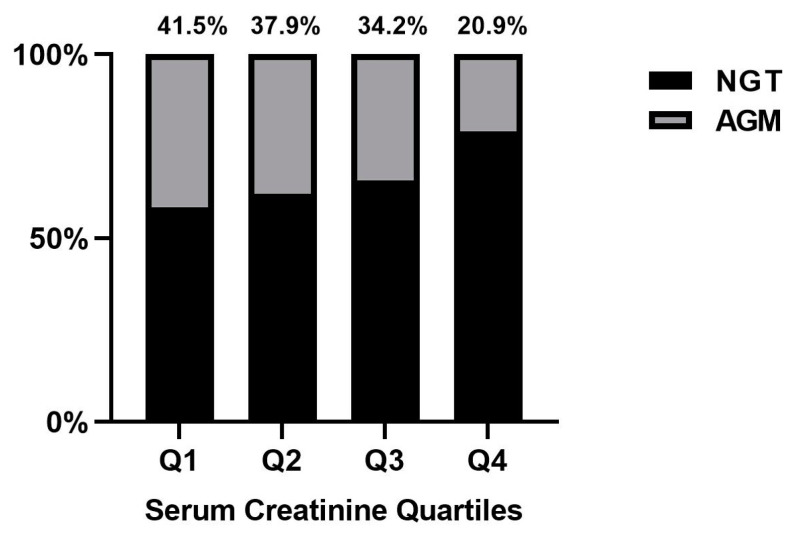
The prevalence of postpartum AGM according to first-trimester serum creatinine quartiles. Abbreviations: NGT stands for normal glucose tolerance; AGM stands for aberrant glucose metabolism.

Among the 501 women with GDM included in the analysis, 170 (33.9%) women developed postpartum AGM, of whom 3.8% had DM, 30.1% had IGT, and no women had isolated IFG. Figure 1 indicates the incidence of postpartum AGM (including IGT and DM) in different serum creatinine quartiles. The lowest incidence of postpartum AGM was observed in the highest quartile of serum creatinine, with only 20.9% of women developing the condition, compared to 41.5%, 37.9%, and 34.2% in the lowest to third quartile, respectively (Chi-square test, *p* = 0.007).

Table 2 presents crude and adjusted ORs of postpartum AGM by baseline creatinine quartiles. The incidence of postpartum AGM increased with decreasing serum creatinine quartiles, with the highest incidence found in the lowest quartile. In the unadjusted model, lower quartiles were significantly correlated to an increased danger of postpartum AGM (OR 2.69 [95% CI 1.49–4.83], 2.31 [95% CI 1.29–4.15] and 1.97 [95% CI 1.13–3.48], respectively) than the highest quartile. Upon adjusting for maternal age, postpartum AGM was 3.18 times more likely to occur in women in the lowest creatinine quartile than in those in the highest creatinine quartile (OR 3.18 [95% CI, 1.74–5.82]; *p* < 0.001). Model 2, which accounted for maternal age, pregestational BMI, family history of T2DM, and preceding history of GDM, demonstrated that lower creatinine quartiles were still significantly correlated to postpartum AGM. In the fully adjusted model, which included pregnancy age, pregestational BMI, family history of T2DM, preceding history of GDM, gain in pregnancy weight, insulin use, fasting glucose at 10–14 weeks and glucose level 120 min after the OGTT during 24–28 weeks of pregnancy, lower quartiles were still related to a higher risk of postpartum AGM (adjusted OR 3.37 [95% CI 1.77–6.42], 2.42 [95% CI 1.29–4.51] and 2.27 [95% CI 1.23–4.18], respectively) than the highest quartile.

### 3.3. Association of Serum Creatinine and Postpartum AGM Incidence: Subgroup Analyses

For further analysis, we stratified the women by maternal age (median age of 33 years), BMI (overweight is BMI ≥ 23 kg/m^2^) (according to Asia-specific standards from the WHO), and dyslipidemia. Stratified analysis revealed consistent significant associations between serum creatinine and postpartum AGM across all subgroups. The fully adjusted model demonstrated that women in the lowest creatinine quartile are exposed to an increased risk of postpartum AGM by comparison to those in the highest creatinine group in all subgroups. The middle two quartiles were linked to a higher potential of postpartum AGM, only in the higher age group, the lower BMI group, and the dyslipidemia group (Table 3).

### 3.4. Association of Continuous Serum Creatinine Levels and Postpartum AGM Incidence

**Figure 2:**
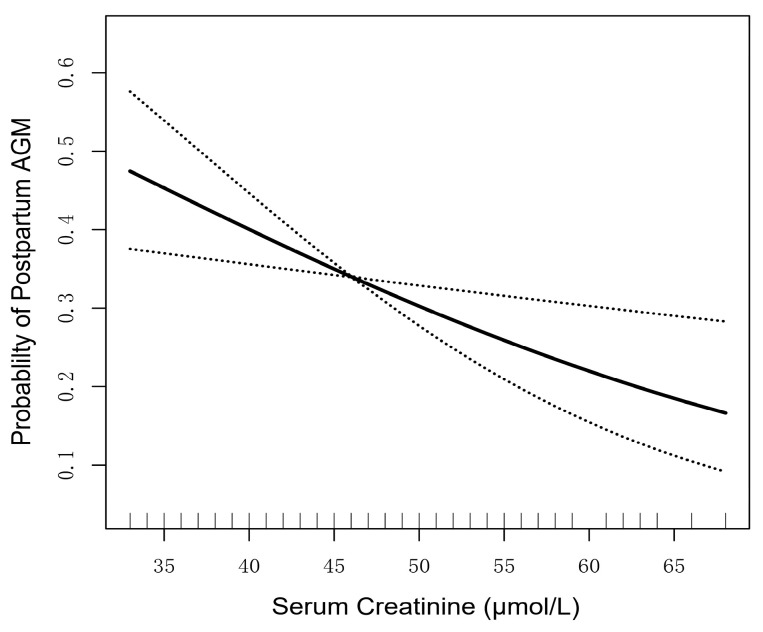
Association between continuous creatinine and the risk of postpartum AGM, using the generalized additive mixed model. The solid line depicts the smooth curve fit between variables. Dotted lines refer to the 95% confidence interval from the fit.

A generalized additive model (GAM) was utilized to build a smooth curve and detect the relationship between serum creatinine and the risk of postpartum AGM. In light of the unadjusted GAM analysis, a linear correlation was observed between serum creatinine levels and postpartum AGM risk below a level of 68 µmol/L (upper limit in the women in our study) and the risk of postpartum AGM (Figure 2).

Considering serum creatinine level as a continuous variate (Table 4), the logistic regression model revealed that each 2 μmol/L decrease in serum creatinine was linked to 8% higher odds of postpartum AGM below a serum creatinine threshold of 68 µmol/L at 10–14 weeks gestation in women with GDM. Similarly, in terms of the age-adjusted model as well as the fully adjusted model, the logistic regression model revealed that higher levels were linked to a decreased incidence of postpartum AGM (*p* = 0.001, *p* = 0.003, respectively). Findings from the adjusted model indicated that a decline of 2 µmol/L in serum creatinine was significantly associated with a 10% rise in the likelihood of postpartum AGM. 

### 3.5. Continuous Serum Creatinine Levels and Postpartum Glucose Level, β-Cell Function and HOMA-IR

Calculation of β-cell function was performed, applying data from the OGTT: IGI (Δinsulin (30–0 min)/Δglucose (30–0 min)). Insulin resistance was quantified by means of HOMA-IR. Linear regression was employed to investigate the correlations among log-transformed serum creatinine and log-transformed postpartum fasting glucose, 2-h glucose, IGI, and HOMA-IR (Table 5). The investigation of linear regression indicated that the serum creatinine level was negatively associated with postpartum 2-h glucose level and positively linked to IGI. In the multivariate model, a high serum creatinine level was associated with a lower postpartum 2-h glucose level (β = −0.23, 95% CI [−0.40, −0.07], *p* = 0.007) and a better IGI (β = 0.55, 95% CI [−0.06, 1.04], *p* = 0.027). In adjusted models, the serum creatinine level did not correlate with FPG or HOMA-IR.

## 4. Discussion

In the current retrospective cohort analysis of women who have recently experienced GDM, we found that low serum creatinine levels at 10–14 weeks gestation were positively associated with an increased risk of postpartum AGM incidence, regardless of other major risk factors such as age, BMI, gestational weight increase, family history of T2DM, previous history of GDM, insulin use and glucose level. Additionally, analysis using the unadjusted GAM indicated that serum creatinine and the risk of postpartum AGM are correlated linearly. In the adjusted model, postpartum AGM risk decreased by 10% for every 2 μmol/L increase in serum creatinine in patients with GDM who had serum creatinine levels below 68 µmol/L. 

Serum creatine, generally considered an easily measured surrogate marker of skeletal muscle mass, has reportedly been linked to the occurrence of T2DM. N. Harita et al. first reported the link between T2DM occurrence and serum creatinine in their 4-year cohort investigation of 8570 Japanese male, revealing that lower levels of serum creatinine independently predicted progression to T2DM in nonobese Japanese men with normal creatinine levels at baseline [27]. Since then, a further cohort research study in an eastern Asian community shed light on a link between low blood creatinine levels and a higher prevalence of diabetes in both men and women, with a reverse J-shaped correlation in males and a linear relationship in women [29]. Interestingly, a cross-sectional study in Caucasian women also showed a piecemeal linear correlation of T2DM and serum creatinine, indicating that the risk of T2DM was 6% lower in women with a serum creatinine level below 69 µmol/L for every 1 mol/L increase in serum creatinine, which resembles the findings of our study [35]. 

Our findings extended this association specifically to pregnant women initially, providing evidence for a correlation among serum creatinine and persistent glucose metabolism disorders in women with GDM. The serum creatine level at the first antenatal visit, measured in a simple and rapid blood test, might be a potential biomarker for postpartum AGM among women with GDM. As far as we are aware, one study examined metabolites in the first trimester and found that, by comparison to women with normal glucose tolerance (NGT), those who subsequently developed GDM had higher levels of anthranilic acid, glutamate, alanine, and allantoin, along with significantly lower levels of creatinine [36]. This indirectly underpins our finding that lower levels of creatinine in early pregnancy may be linked to a higher risk of metabolic abnormalities. It would be worthwhile to further explore whether low serum creatinine levels are associated with an increased incidence of GDM. 

The underlying mechanism by which lower serum creatinine levels increase the risk of postpartum AGM remains unknown in this study. However, it is possible that lower serum creatinine levels indicate decreased skeletal muscle mass, as hypothesized. In our study, women with GDM had a median pre-pregnancy BMI of just 21.5 kg/m^2^, and we discovered that early postpartum glucose metabolism disorder in these women primarily manifested as IGT rather than IFG, which is consistent with previous Asian studies [17,18,37]. Unlike individuals with IFG who are generally obese, accompanied by hepatorenal insulin resistance and elevated gluconeogenesis, individuals with IGT have reduced peripheral insulin sensitivity (mainly representing skeletal muscle), combined with impairments in glucose-stimulated insulin secretion, resulting in postprandial hyperglycemia [38,39,40]. Skeletal muscle serves as a key player as the major site of postprandial glucose disposal, accounting for about 85% of glucose uptake in the postprandial state [19]. Accordingly, the increased risk of glucose intolerance associated with low serum creatinine levels may be explained by low muscle mass.

Our current study demonstrated a negative association between low serum creatinine and postpartum β-cell function, but we did not observe any association between serum creatinine and HOMA-IR, which is a measure of hepatic insulin resistance. It is important to note that individuals with impaired glucose tolerance typically exhibit muscle insulin resistance with only mild hepatic insulin resistance. Future studies should explore muscle insulin sensitivity using the hyperinsulinemic-euglycemic clamp technique. Among young women, the desire to shed weight is very common; however, inadequate muscle mass might be a potential cause for the inability to maintain normal glucose tolerance and may lead to persistent glucose abnormalities in GDM women, suggesting that aerobic and resistance exercise before and after pregnancy may contribute to the prevention of postpartum diabetes by increasing muscle mass and improving β-cell function, which should be further confirmed by prospective and multicenter studies.

Numerous variables, most notably deteriorating renal function, might affect serum creatinine levels. In this study, we excluded women with underlying kidney injury or other conditions that could affect serum creatine levels, such as prediabetes, hypertension, and connective tissue diseases, to minimize the effect of other factors on creatinine. The concentration of serum creatinine is influenced by age, gender, and body composition because creatinine is produced from muscle tissue. In this study, we adjusted for these factors and still observed a significant correlation between serum creatine and postpartum glucose disorders. In addition to the abovementioned factors, serum creatinine is also affected by nutritional status and protein intake [41]. There is evidence to suggest that dietary factors, particularly protein and the amino acid leucine, play a critical role in promoting muscle protein synthesis and preserving muscle mass [42,43]. Vegetarians have considerably lower serum creatinine concentrations than omnivores in numerous big cross-sectional investigations [44,45]. Interestingly, an animal study revealed that insufficient nutrition during the first half of pregnancy is associated with later metabolic abnormalities and favors a diabetogenic condition in the pregnant mother [46]. Further investigation is required to determine if early nutrition is related to the increased risk of glucose abnormalities associated with low serum creatinine levels. Additionally, it is important to note that glomerular hyperfiltration, which is linked to lower serum creatinine levels, has been shown to raise the risk of metabolic disorders, including diabetes [47,48]. Potential mechanisms include insulin resistance, as insulin has been shown to exert a direct effect on glomerular podocytes [47], increased renal gluconeogenesis [49], and endothelial dysfunction/chronic inflammation [50]. Serum creatinine levels also typically decrease during pregnancy due to physiologic glomerular hyperfiltration [51], while higher glomerular filtration rates have been found to increase the risk of adverse maternal/fetal outcomes [52,53,54]. Our study showed a potential association between low serum creatine in early pregnancy and persistent glucose metabolism disorders after adjusting for age, and higher glomerular filtration rates may also be a factor. However, the correlation between glomerular filtration rate and creatinine level makes it difficult to determine which is the culprit.

The reliability and validity of our findings are strengthened by the following: the serum creatinine levels were assessed during the initial antenatal visit, and prior to detecting any glucose abnormalities, women with underlying kidney disease or other conditions that might affect serum creatinine levels were excluded, the models were adjusted for multiple confounders, and standardized criteria were used in our universal screening and diagnosis. Therefore, in spite of our study’s retrospective nature, this research is still of great value in demonstrating the correlation between low serum creatinine concentration and the progression of postpartum AGM. Nonetheless, the present study is not exempt from certain limitations. First, it would be preferable to have pre-pregnancy creatinine data; however, because pregnancies can sometimes be unintended, these data are challenging to collect. Prospective studies among fertile women can thus be conducted in the future. In addition, the actual measured skeletal muscle mass was not analyzed in this study; thus, more far-reaching research is demanded to shed light on mechanisms underlying our findings.

## 5. Conclusions

The findings of this investigation shed new light on the link between low serum creatinine levels in early pregnancy and the increased incidence of postpartum AGM, suggesting that low serum creatine levels at the first antenatal visit, measured in a simple and rapid blood test, might be a potential biomarker for postpartum AGM among women with GDM. The inverse correlation between serum creatinine and persistent glucose metabolism disorders might reflect the protective effect of skeletal muscle on glucose metabolism. Further research is required to understand the mechanisms underlying our findings, as well as the role of skeletal muscle mass or nutritional status in early pregnancy on later glucose metabolism.

## Figures and Tables

**Table 1:** Characteristics of the women in this study based on serum creatinine quartiles at the first antenatal visit.

	Total Sample	Serum Creatinine				*p* Value
Characteristics		Q1 (33–41 µmol/L)	Q2 (42–45 µmol/L)	Q3 (46–50 µmol/L)	Q4 (51–68 µmol/L)	
n	501	118	124	149	110	
Maternal age (years)	33 (30–37)	33 (29–35)	33 (30–37)	33 (30–37)	35 (31–38.3)	0.009
Height (cm)	159 (156–163)	158 (155–161)	158 (155–161)	160 (156–164)	160 (157–164)	0.005
Weight before pregnancy (kg)	54 (50–60)	53 (48–58)	54 (50–60)	54 (50–61)	56 (52–62)	0.030
Pre-pregnancy BMI (kg/m^2^)	21.5 (19.8–23.4)	21.2 (19.3–23.2)	21.7 (20.0–23.5)	21.1 (20.0–23.4)	22.7 (20.2–23.8)	0.155
Weight delivery	65.0 (60.0–70.7)	63.0 (59.7–69)	65.5 (60–70)	65.6 (60.1–73.2)	65.5 (61–71.1)	0.090
Gestational weight gain (kg)	10.0 (7.5–13.2)	10.5 (7.5–13.4)	10.0 (7.4–12.7)	11.0 (8.3–14.5)	9.5 (7–12.2)	0.095
Previous GDM, n (%)	20 (4)	5 (4.2)	6 (4.8)	6 (4)	3 (2.7)	0.871
Family history of T2DM, n (%)	84 (16.8)	6 (11.8)	2 (4.7)	9 (19.6)	3 (7.3)	0.403
Insulin-requiring GDM, n (%)	7 (1.4)	2 (1.7)	1 (0.8)	3 (2)	1 (0.9)	0.808
FPG (mg/dL)	77.4 (72–82.8)	76.5 (72–82.8)	77.4 (72–82.8)	77.4 (72–82.8)	77.4 (71.6–82.8)	0.982
ALT (U/L)	13.0 (10.0–18.0)	12.0 (9.0–18.3)	13.0 (10.0–18.0)	13.0 (10.0–18.0)	13 (10–19)	0.512
AST (U/L)	17.0 (15.0–20.0)	17.0 (15.0–21.0)	17.0 (16.0–20.0)	18.0 (15.0–21.0)	17.0 (15.0–20.0)	0.758
GGT(U/L)	13.0 (11.0–16.3)	13.0 (10.0–18.0)	13.0 (10.0–17.0)	13.0 (11.0–16.0)	12.0 (10.0–16.0)	0.611
BUN (mmol/L)	2.7 (2.2–3.2)	2.5 (2.1–2.9)	2.7 (2.3–3.1)	2.7 (2.4–3.1)	3.1 (2.7–3.5)	<0.001
24–28 w FPG (mg/dL)	81.0 (75.6–86.4)	81.0 (75.6–86.4)	81.0 (75.6–86.4)	81.0 (75.6–86.4)	79.2 (75.6–88.2)	0.963
24–28 w 1-h glucose (mg/dL)	180 (165.6–190.8)	180 (163.8–194.4)	180 (169.2–192.6)	178.2 (163.8–192.6)	180 (169.2–189)	0.680
24–28 w 2-h glucose (mg/dL)	158.4 (151.2–171)	160.2 (147.6–171)	160.2 (151.2–172.8)	158.4 (149.4–169.2)	158.4 (153–171)	0.615
24–28 w TG (mmol/L)	2.1 (1.8–2.7)	2.1 (1.9–2.7)	2.1 (1.8–2.6)	2.1 (1.7–2.7)	2.2 (1.8–2.7)	0.802
24–28 w LDL-C (mmol/L)	3.4 (3.0–4.0)	3.4 (2.9–4.0)	3.5 (3.1–4.1)	3.4 (3.0–3.9)	3.5 (3.0–4.1)	0.486
24–28 w HDL-C (mmol/L)	2.0 (1.7–2.3)	1.9 (1.6–2.2)	2.0 (1.7–2.2)	2.0 (1.7–2.3)	2.0 (1.8–2.2)	0.441

Note: data are expressed as the mean ± SD, median (IQR) or n (%). Abbreviations: GDM, gestational diabetes mellitus; BMI, body mass index; FPG, fasting plasma glucose; AST, aspartate transaminase; ALT, alanine aminotransferase; GGT, γ-glutamyl transpeptidase; BUN, blood urea nitrogen; TGs, triglycerides; LDL-C, low-density lipoprotein cholesterol; OGTT, oral glucose tolerance test; HDL-C, high-density lipoprotein cholesterol.

**Table 2:** Association between serum creatinine quartiles in early pregnancy and postpartum AGM.

	Unadjusted Model		Model 1		Model 2		Model 3	
	OR (95 % CI)	*p*	OR (95 % CI)	*p*	OR (95 % CI)	*p*	OR (95 % CI)	*p*
Q1 (33–41μmol/L)	2.69 (1.49–4.83)	0.001	3.18 (1.74–5.82)	<0.001	2.98 (1.61–5.50)	0.001	3.37 (1.77–6.42)	<0.001
Q2 (42–45μmol/L)	2.31 (1.29–4.15)	0.005	2.55 (1.41–4.63)	0.002	2.45 (1.34–4.49)	0.004	2.42 (1.29–4.51)	0.006
Q3 (46–50μmol/L)	1.97 (1.11–3.48)	0.020	2.18 (1.74–5.82)	0.008	2.04 (1.13–3.68)	0.018	2.27 (1.23–4.18)	0.008
Q4 (51–68μmol/L)	Reference		Reference		Reference		Reference	

Note: Model 1 adjusted for maternal age. Model 2 adjusted for maternal age, pregestational BMI, family history of T2DM, and preceding history of GDM. Model 3 adjusted for factors including maternal age, pregestational BMI, family history of diabetes, previous history of GDM, gestational weight increase, insulin use, fasting glucose levels during 10–14 weeks of pregnancy and glucose level 120 min following the OGTT during 24–28 weeks of pregnancy.

**Table 3:** Association of serum creatinine and postpartum AGM incidence: subgroup analyses.

	Serum Creatinine						
	Q1 (33–41 µmol/L)		Q2 (42–45 µmol/L)		Q3 (46–50 µmol/L)		Q4 (51–68 µmol/L)
	OR (95%CI)	*p* Value	OR (95%CI)	*p* Value	OR (95%CI)	*p* Value	
Maternal age (years)							
<median	3.29 (1.19–9.13)	0.022	1.44 (0.51–4.04)	0.493	2.56 (0.96–6.81)	0.060	Reference
≥median	3.96 (1.69–9.28)	0.002	3.41 (1.52–7.66)	0.003	2.07 (0.93–4.60)	0.076	Reference
BMI (kg/m^2^)							
<23	2.53 (1.18–5.43)	0.017	2.28 (1.08–4.80)	0.030	2.26 (1.11–4.62)	0.025	Reference
≥23	7.44 (2.10–26.32)	0.002	2.86 (0.86–9.51)	0.087	2.23 (0.63–7.92)	0.216	Reference
Dyslipidemia							
Yes	6.15 (1.49–25.33)	0.012	6.16 (1.61–23.55)	0.008	4.13 (1.02–16.74)	0.047	Reference
No	3.02 (1.39–6.55)	0.005	1.58 (0.74–3.37)	0.240	1.84 (0.89–3.81)	0.099	Reference

Note: adjusted factors included maternal age, pregestational BMI, family history of T2DM, preceding history of GDM, gestational weight increase, insulin use, fasting glucose at 10–14 weeks of gestation and glucose level 120 min after the OGTT at 24–28 weeks of gestation. OR: odds ratio, CI: confidence interval. BMI: body mass index.

**Table 4:** Continuous serum creatinine levels and postpartum AGM incidence.

	Crude		Adjusted For age		Multiple Adjusted	
	OR (95 % CI)	*p* Value	OR (95 % CI)	*p* Value	OR (95 % CI)	*p* Value
Serum creatinine (Per 2 μmol/L increase)	0.92 (0.86–0.98)	0.007	0.90 (0.85–0.96)	0.001	0.90 (0.84–0.96)	0.003

Note: multiple adjusted for variables included: maternal age, pregestational BMI, family history of T2DM, preceding history of GDM, gestational weight increase, insulin use, fasting glucose at 10–14 weeks of gestation and glucose level 120 min after the OGTT at 24–28 weeks of gestation.

**Table 5:** Continuous serum creatinine levels and postpartum glucose level, β-cell function, and HOMA-IR.

	Crude		Adjusted For Age		Multiple Adjusted	
	β (95 % CI)	*p* Value	β (95 % CI)	*p* Value	β (95 % CI)	*p* Value
FPG (mmol/L)	0.07 (0. 001–0.14)	0.049	0.05 (0.97–0.96)	0.154	0.05 (−0.01 −0.12)	0.104
2-h glucose (mmol/L)	−0.17 (−0.34–0.01)	0.040	−0.22 (−0.39–0.06)	0.009	−0.23 (−0.40–0.07)	0.007
Insulinogenic index	0.54 (0.05–1.03)	0.030	0.62 (0.13–1.11)	0.014	0.55 (0.06–1.04)	0.027
HOMA-IR	0.33 (−0.08–0.75)	0.117	0.04 (−0.02–0.10)	0.180	0.24 (−0.15–0.63)	0.235

Note: linear regression to assess associations of log-transformed serum creatinine with log-transformed postpartum fasting glucose, 2-h glucose, insulinogenic index, and HOMA-IR. Multiple adjusted for variables including: maternal age, pre-gestational BMI, family history of T2DM, preceding history of GDM, gestational weight increase, insulin use, fasting glucose at 10–14 weeks and glucose level 120 min after OGTT at 24–28 weeks of gestation.

## Data Availability

The data can be retrieved from the corresponding author upon reasonable request.
